# Contributions of Basic Cognitive Processing to Chinese Reading: The Mediation Effect of Basic Language Processing

**DOI:** 10.3389/fpsyg.2018.02670

**Published:** 2019-01-08

**Authors:** Xiujie Yang, Peng Peng, Xiangzhi Meng

**Affiliations:** ^1^Department of Psychology, The Chinese University of Hong Kong, Hong Kong, China; ^2^Department of Special Education, The University of Texas at Austin, Austin, TX, United States; ^3^School of Psychological and Cognitive Sciences, Beijing Key Laboratory of Behavioral and Mental Health, Peking University, Beijing, China

**Keywords:** kindergarten children, Chinese reading, basic cognitive processing, basic language processing, mediation effect

## Abstract

Prior research has mostly focused on either basic language or basic cognitive precursors of reading development, but relatively little is known about their relative importance for reading, especially for Chinese beginning readers. The present study examined whether and how basic cognitive processing (executive function, attention, and visual-spatial perception) and basic language processing (phonological awareness, morphological awareness, orthographic awareness, and RAN) measured at kindergarten influence Chinese character reading and reading comprehension in the first grade. Results showed that basic language abilities including morphological awareness and rapid automatized naming predicted later Chinese character reading. Only one basic cognitive skill, sustained attention, predicted later reading comprehension. Mediation analysis showed that the overall effects of basic cognitive skills on later character reading and reading comprehension were mediated by basic language skills. These findings supported an integration reading model for early Chinese reading and basic language processing at kindergarten plays an important role in explaining the relation between basic cognitive processing and grade one reading performance.

## Introduction

The aim of the current study is to investigate the longitudinal contributions of basic cognitive and basic language factors to early reading. According to the Simple View of Reading, reading includes two basic components: word reading (decoding) and reading comprehension (Gough and Tunmer, 1986; Hoover and Gough, 1990; Silverman et al., 2013). Given that children have a high risk of reading problems, and quite a few children need interventions to alleviate potential reading difficulties, it is very important to understand the underlying factors that influence reading acquisition as early as possible. However, effects of basic cognitive and basic language skills on word reading and reading comprehension were mostly investigated separately and research rarely focused on early readers in the past decades (e.g., McBride-Chang et al., 2008; Meng et al., 2011).

Currently, there is a debate on whether and how basic cognitive and basic language abilities influence reading acquisition (Wagner and Torgesen, 1987; Cain et al., 2004; Seigneuric and Ehrlich, 2005; Yang et al., 2013; Brandenburg et al., 2017; Kim, 2017). One view is focused on the role of basic cognitive factors in reading (Cain et al., 2004; Seigneuric and Ehrlich, 2005; Yang et al., 2013). For example, some research suggests basic cognitive skills such as visual-spatial skills, working memory and executive functions develops early on and thus facilitate reading development (Cain et al., 2004; Seigneuric and Ehrlich, 2005; Yang et al., 2013; Peng et al., 2018). In contrast, some argued that basic language abilities are more important for reading. For instance, research repeatedly showed that meta-linguistic skills such as phonological awareness and RAN are critical for reading in alphabetic languages such as English (e.g., Wagner and Torgesen, 1987) and German (e.g., Brandenburg et al., 2017), while morphological awareness are critical for Chinese reading (Shu et al., 2006; Tong et al., 2009).

Moreover, an increasingly popular view posits an integration model emphasizing that both cognitive and language skills are important in the reading process (e.g., Peng et al., 2016; Kim, 2017). For example, Kim (2017) found that for English-speaking second-graders, working memory, vocabulary, grammar, and inference had direct influence on word reading and reading comprehension; Working memory could also indirectly influence reading comprehension through language skills. The present study aims to take the integration approach to investigate the whether and how basic cognitive and basic language influence early Chinese reading. In comparison to Kim (2017), we focused on early Chinese reading and included a more comprehensive range of basic cognitive and basic language skills.

### Basic Cognitive Processing and Reading

Basic cognitive abilities play important roles in learning across academic domains, including reading, writing, and mathematics (Fuchs et al., 2016; Peng et al., 2016). Working memory, inhibition and attention of kindergarteners or preschoolers are important predictors of later reading abilities (McClelland et al., 2006; Walcott et al., 2009; Lan et al., 2011; Lee et al., 2013) and reading comprehension (Brock and Knapp, 1996; Follmer, 2018). Specifically, during reading, working memory is needed to process target stimuli while inhibitory control is needed to prevent influences of irrelevant information (Engle et al., 1999). So, working memory might be both important in reading comprehension (Daneman and Carpenter, 1980) and word reading (Verhoeven et al., 2011). Additionally, sustained attention, one of the most important elements of attention, is essential for maintaining the simultaneous activation of the target characters (Barrouillet et al., 1997), and take effects on reading comprehension (Daneman and Carpenter, 1980). Lam and Beale (1991) showed primary children’s performance in the sustained task was significantly associated with reading achievement. However, these children were selected from elementary schools where they had been educated by formal reading. Little is known about the role of sustained attention during the early stage of word reading and reading comprehension.

An additional general processing ability is visual spatial perception. Visual spatial perception is rarely addressed in reading (Siok and Fletcher, 2001). However, some children with developmental dyslexia have shown deficits in motion perception and higher coherent motion thresholds (Meng et al., 2014), and some poor readers have manifested problems in visual attention, oculomotor control, and spatial position encoding (Boden and Giaschi, 2007; Goswami, 2015). Especially for Chinese characters, which generally consist of more strokes than English, the visual representation is quite complicated. In addition, because Chinese characters lack reliable grapheme-phoneme correspondences, the visual spatial information of character structure might be particularly important for Chinese readers, as compared to, for example, readers of English (Chen et al., 2003; Chung et al., 2010; Wang et al., 2015). Because character recognition is involved during reading comprehension, we also included visual spatial perception as a potential predictor of reading comprehension.

### Basic Language Processing and Reading

In addition to general abilities, children make use of basic language processing for reading. Previous studies have highlighted several correlates of Chinese reading, including phonological awareness (Siok and Fletcher, 2001; Chow et al., 2005; Shu et al., 2008; Pan et al., 2011; Xue et al., 2013; Pan and Shu, 2014), morphological awareness (Ku and Anderson, 2003; McBride-Chang et al., 2005; Liu and McBride-Chang, 2010; Lei et al., 2011), orthographic awareness (Lonigan et al., 2000; Ho et al., 2003; Ho et al., 2004; Levy et al., 2006; Wang et al., 2015), and rapid automatized naming (RAN; Denckla and Rudel, 1976; Badian, 1993; Kirby et al., 2003; Swanson and Hammill, 2003; Savage and Frederickson, 2005). For example, orthographic awareness explained unique variance in word reading (operationally defined as one single character) (Wang and McBride, 2016). In another study, McBride-Chang et al. (2008) found that children at risk for dyslexia performed significantly worse only on tone detection, an aspect of phonological sensitivity, and morphological awareness compared to typically developing peers. In contrast, Wang et al. (2015) found that one unique correlate of Chinese character reading was RAN for kindergarteners. Tong and McBride-Chang (2010) categorized phonological awareness, morphological awareness, and orthographical awareness as the metalinguistic awareness for Chinese reading. Because RAN is the core deficit in reading disability (Norton and Wolf, 2012), we also included it as the basic language process of reading.

### The Mediation Hypothesis of Basic Language Processing

How do the basic cognitive and basic language processing affect children’s reading? Previous studies showed basic cognitive abilities, including executive function, visual spatial abilities, and perceptual sensitivity, could affect the development of reading performance via basic language processing (Eden et al., 1996; Talcott et al., 1999; Tallal, 2004; Conlon and Sanders, 2011; Franceschini et al., 2012; Olulade et al., 2013; Boros et al., 2016; Chung et al., 2016; Kim, 2017). For example, Chung et al. (2016) have found that executive function made indirect significant contributions to reading through phonological awareness. Meng et al. (2011) demonstrated that dynamic visual perception could be specifically related to orthographic processing in reading Chinese characters. Finally, some Chinese studies replicated the direct effects of basic language processing on reading acquisition of young children (e.g., Lin et al., 2010). We therefore proposed the mediation hypothesis in the integration model to explore the mediation effect of basic language processing on the relation between basic cognitive abilities and Chinese character reading and reading comprehension.

### The Present Study

Effects of basic cognitive and basic language skills on reading have mostly been investigated separately (Lei et al., 2011; Christopher et al., 2012; Berninger et al., 2016; Guajardo and Cartwright, 2016), and little research has investigated their relative importance in early Chinese reading simultaneously. Moreover, the reading process in previous studies was mostly measured by only character reading (e.g., McBride-Chang et al., 2008). But it should be noted reading should involve both word recognition and comprehension (Perfetti, 1999). To fill the gap, the present study will examine whether and how basic cognitive processing and basic language processing measured at kindergarten influence Chinese character reading and reading comprehension at first grade. First, we will explore the unique contribution of basic cognitive (working memory, inhibition and attention) and basic language skills (phonological awareness, morphological awareness, orthographic awareness, and RAN) to Chinese character reading and reading comprehension. Second, we will examine whether basic language skills mediate the relation between basic cognitive skills and Chinese character reading and reading comprehension. Given that comprehension and character reading are correlated but separable, some cognitive precursors will uniquely predict comprehension, while others may be critical to character reading. Basic language skills are usually shown very important in character reading (Li et al., 2012). Basic cognitive skills such as sustained attention and working memory have shown very important in the reading comprehension (Brock and Knapp, 1996; McVay and Kane, 2012). Thus, we predict that basic language skills might show closer relations with character reading, while basic cognitive skills would show closer relation with reading comprehension.

Early Chinese reading predictors should be somewhat different from alphabetic language like English or similar-structured language like Korean considering their differences (Taylor and Taylor, 2014). The logography and orthography in Chinese characters make it easier to grasp the meaning than alphabetic languages (Shu et al., 2006). For example, “
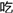
”/chi1/ means eating, while the radicle “
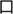
”/kou2/ in it means the mouth. But English is more phonetically predictable with 26 letters. Compared to alphabetic languages, the phonetic is not always reliable of providing the character’s meaning or sound, making it very difficult for Chinese-speaking children to master it (Zhou, 1980; Shu et al., 2003). In Korean, the structure of Korean Hangul is combined with phonemes, and requires children to have some sense of phoneme onsets, as well as syllables, to become fluent readers. Therefore, compared to English and Korean, phonological skills are not fully supported in early Chinese reading instruction (McBride-Chang et al., 2005). Moreover, there are many homophones that sound the same, but the meanings are different in Chinese. For example, /gan1/ can be /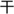/ (“dried”) or /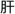/ (“liver”). Children need to distinguish what is the exact meaning of /gan1/ in the context and inhibit another /gan 1/, especially for Chinese young beginning learners (Lin et al., 2010; Chung and McBride-Chang, 2011; Peng et al., 2013). Thus, meaning-related morphological awareness might be therefore more important to Chinese reading compared to alphabetic languages (McBride-Chang et al., 2005; Shu et al., 2006). Korean, whose morphological structures are like Chinese scripts, was also found morphological awareness was very important when acquiring reading abilities (McBride-Chang et al., 2005).

## Materials and Methods

### Participants

Participants were 108 Mandarin-speaking children (63 males) who were recruited from two kindergartens in a northern city of China and were tested first in the fall semester of their last year in Kindergarten (time 1–T1) and then in the spring semester of their first year in primary school. The children were reported by their parents with no developmental disorders or developmental retardation. Among these children, 82 children (46 males) completed all testing at both T1 and T2 (time 2–T2), whereas 26 children did not participate at T2 because they transferred to other schools. According to the teachers’ report, children in our study mostly have Beijing *hukou* (registered permanent residence), and their parents have college diploma with regular earnings. All the children were from middle-class families accordingly. The average age of the children at T1 was 68 months (*SD* = 0.30) and at T2 was 82 months (*SD* = 0.35). To assess the problem of longitudinal attrition, we performed a series of independent *t-*test for the two independent samples (i.e., 82 retained children and 26 transferred/lost children) in all measurements. None reached significance (all *p*s > 0.05), suggesting that children who transferred did not differ significantly from those who did not in terms of their performances. The study procedure was approved by the Ethics Committee of Peking University. All subjects gave written informed consent in accordance with the Declaration of Helsinki. Within our ethics statement, the consent obtained from the parents of all research participants was both informed and written.

### Procedure

Character reading was assessed at kindergarten and grade one. Listening comprehension, general cognitive measures, including executive function, sustained attention, and visuospatial perception and basic language skills, including phonological awareness, morphological awareness, orthographic awareness, rapid automatized naming, were assessed at kindergarten. Because reading comprehension task is too difficult for kindergarten children, we only assessed it at grade one. Testing at time 1 involved three sessions and each session lasted no more than 45 min to reduce fatigue. Children finished the assessments in three consecutive days. At grade one, only two tasks, character reading and reading comprehension, were administered, and children completed them in one session. All tasks were randomly administered.

### Measures

#### Basic Cognitive Measures

##### Executive function

According to Lee et al. (2013), the components of executive function for kindergarten children include working memory and inhibition. To evaluate children’s working memory, we administered the forward and backward digit span task from the Wechsler Intelligence Scale-IV (Wechsler, 2008) and the visuo-spatial sketchpad test (Baddeley, 2003). For the forward digit span task, the tester read several digits aloud at a rate of one digit per second and then asked the participant to repeat the digit lists in the same sequence. The test began with a three-digit length and progressed to a ten-digit length with two lists at each length. If both lists were correctly repeated, the length was increased by one more digit, and another two lists were administered. If the child failed twice in succession, testing stopped. Participants’ final performance was the total numbers of lists they could replicate correctly. The Cronbach’s alpha for the current sample was 0.70. For the backward digit task, the tester read a series of digits aloud at a rate of one digit per second and then asked the child to recall the reverse order of digits in sequence. Digit spans ranged in length from two to nine. Each span included two series of digits of the same length. The test stopped when the child failed to recall two lists in succession. The final score was the total number of lists recalled correctly. The Cronbach’s alpha for the current sample was 0.60. Lastly, the visuospatial sketchpad test measures children’s ability to remember visual sequences within a matrix (Lezak et al., 2004; Holmes et al., 2008). In this task, a little mouse tapped from three to seven blocks within a matrix, and the child duplicated the sequence which the mouse tapped. The test stopped when the child failed to recall two sequences consecutively. The total score was the number of sequences recalled correctly. The Cronbach’s alpha for the current sample was 0.85.

**FIGURE 1:**
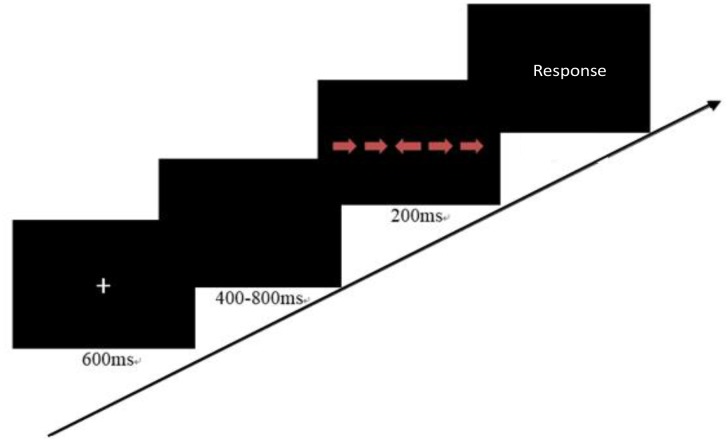
A trial of the adapted Flanker task.

To assess children’s inhibition, we used an adapted Flanker Task (Ridderinkhof and van der Molen, 1995). For each trial, five arrows were shown on the screen in a line. Children needed to judge the direction the center arrow pointed to. In the incongruent condition, the direction of arrows around the target arrow was contrary to the target central one. In the congruent condition, all the arrows pointed in the same direction. This task consisted of 10 practice trials and 40 experimental trials. The procedure of each trial is presented in Figure 1. The accuracies and correct reaction times of responses and the percentages of false positives were recorded. The reaction time in the incongruent condition and the accuracy were related, *r* = –0.31, *p* < 0.01. Therefore, the final score for this task was based on the adjustment using the formula: RT × (1+2 × error), in which the error was the error rate in the incongruent condition (Lyons et al., 2014). The Spearman–Brown reliability coefficient was 0.83 for the current sample.

##### Sustained attention

The Continuous Performance Task was used to measure sustained attention (Gardner-Neblett et al., 2014). Sixteen types of figures (squares, circles, triangles, and stars, colored red, yellow, blue, or green) were designed and presented on the screen in the center of the computer screen for 200 ms one at a time. The target stimulus was the red square. The child was asked to press the left arrow key when the target appeared on the screen. There were 10 practice trials and 120 formal test trials. The accuracy and correct reaction time of responses and the percentage of false positives were recorded. As there was a speed-accuracy trade-off between reaction time and accuracy, the final score was adjusted using the same formula RT × (1+2 × error), in which RT stands for correct reaction time and the error means percentage of omission errors (Lyons et al., 2014). The Spearman–Brown reliability coefficient for the current sample was 0.85.

##### Visuospatial perception

We administered the blocks test and the orientation discrimination task to measure visuospatial perception. For the blocks test, the Block Reconstruction subtest from the Chinese version of WPPSI-IV was used (Zhang, 2008). In this test, the patterns were presented with a picture on one sheet or by the tester. Then, the child reconstructed the patterns using blocks within a limited time. The final score was the total number of patterns constructed correctly. The Cronbach’s alpha for the current sample was 0.63.

**FIGURE 2:**
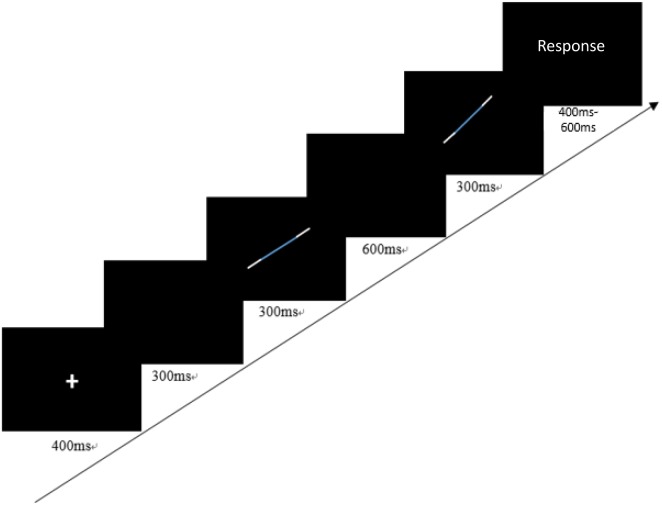
A trial of the orientation discrimination task.

The orientation discrimination task was to assess children’s sensitivity in discriminating differences between two segments’ orientations. It is a two-alternative forced choice task, in which children were asked to determine which direction the second segment turned, compared to the first segment (left/right). The procedure of each trial is presented in Figure 2. On each trial, the order of left and right direction was randomly determined. Children sat 80 cm away from the central screen. The process was designed with 3-down-1-up self-adaptive procedure. The segment was 6° length presented in the first quartile and the step size was 1.0715°. The angle differences between the two segments started from 10.239°and went down when the children answered correctly three times in succession but rose up when the children answered incorrectly once. There were two blocks with 10 reversals in each block. The reversal was defined as the angle between the two segments that rose or decreased. The first two reversal step sizes were two and the last eight reversal step sizes were one. The orientation discrimination threshold was the mean of the angle differences between the two segments for the middle six reversals. The Cronbach’s alpha for the current sample was 0.79.

#### Basic Language Measures

##### Phonological awareness

We administered the syllable deletion task to measure phonological awareness. This task was revised from previous research (McBride-Chang and Ho, 2000; McBride-Chang and Kail, 2002). For the syllable deletion task, children were required to take away one syllable from a two-, three-, or four-character word presented orally by the tester. For example, /zi4 xing2 che1/ without /zi4/ is /xing2che1/. There were 2 practice items and 22 experimental items requiring deleting the first, the second, the third or the last syllable. Half of the items were pseudowords and the other half were real words. The final score was the number of items children correctly answered. The Cronbach’s alpha for the current sample was 0.94.

##### Morphological awareness

This task was revised from previous studies (e.g., McBride-Chang et al., 2008). The experimenter instructed children to combine familiar morphemes in new ways referring to the given examples within the “legal” constraints of the Chinese language. One of the examples was like this: If we call the big and red flower a big red flower, what should we call the small and purple flower? (The correct answer is a small purple flower). There were 3 practice items and 18 test items. Half of the items were noun constructions and the other half were verb constructions. Each correct answer was given two points. Those that included only part of the critical information or that were redundant were given a score of 1 each. The Cronbach’s alpha for the current sample was 0.86.

##### Orthographic awareness

There were three subtests used. The first subtest was to distinguish real characters or digits from reversed ones. We reversed real characters and presented them on the sheet. For example, “
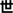
” (the world) was reversed to “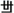.” Sixty characters and 10 digits were included in the assessment. The second subtest required children to identify real characters from non-characters. The real characters were all unfamiliar to the children and therefore children had to distinguish them from non-characters based on orthographic experience. The non-characters were constructed by placing one orthographic radical in an illegal position. Note that the set of orthographic radicals were derived from real character stimuli. This subtest contained 60 characters. The third subtest required children to put the radicals in the proper location to create a real character. The spatial arrangements of each of the orthographic units in a character were either top-bottom or left-right. We chose 19 different radicals, which were familiar to children, from top-bottom and left-right characters. The Cronbach’s alphas for the three subtests were 0.90, 0.73, and 0.75, respectively. The combined Cronbach’s alpha was 0.89.

##### Rapid automatized naming

In this task, children were presented with a sheet of paper with 40 digits on it. The sheet showed five digits (9, 6, 4, 2, and 7) replicated in different orders eight times. Previous studies such as Cui et al. (2017) used a similar measure. To make sure the kindergarten children were familiar with the stimuli, we asked them to name them independently, slowly, first. After that, children were instructed to name all the digits twice, from left to right and from top to down as fast and accurately as possible. The time was recorded by a stop watch. The test–retest reliability for this task was 0.88.

#### Reading Measures

##### Chinese character reading

This test involved 142 characters, divided into ten groups based on frequency level (Wang and Tao, 1993). Children were required to read the Chinese characters aloud within 30 min and skip the unfamiliar ones in grade one. Performance was measured by the total number of characters that children read correctly. Children’s ability of character reading was computed based on the total number of correct characters each participant completed. The Cronbach’s alphas for the current sample were both 0.99 in kindergarten and grade 1.

##### Listening comprehension

This test contained 40 sentences and 5 pictures below each sentence. The experimenter read the sentence for a child, and the child selected the picture that best matched the sentence’s meaning. Considering the difficulty level, the test was administered only in kindergarten. Cronbach’s alpha for the current sample was 0.96.

##### Chinese reading comprehension

In a typical reading comprehension test, children need to read passages with more than two sentences silently, and each passage is followed by several questions (e.g., MacGinitie et al., 2000). However, this kind of assessment was too difficult for young Chinese first graders. Therefore, we revised it into a simpler one. In our test, 95 sentences and 5 pictures below each sentence were provided. Children were asked to read each sentence independently and to select the one that best illustrated its meaning from five pictures. The children were encouraged to complete as many sentences as possible within 10 min. We used this test to evaluate the children’s reading comprehension by calculating the total number of sentences they understood correctly. Considering the difficulty level, the test was administered only at grade 1. Cronbach’s alpha for the current sample was 0.95. The test had been used previously (Meng et al., 2014).

## Results

### Descriptive Statistics

Descriptive Statistics, including the mean scores, standard deviations, skewnesses, kurtosis and reliabilities of each measure, are shown in Table 1. All the assessments had acceptable reliabilities (>0.60). Moreover, most of the variables were normally distributed, except for sustained attention and inhibition. These two variables were logarithmically transformed to improve pairwise linearity and to decrease extreme skewness and kurtosis (Tabachnick and Fidell, 2007). Correlations among compacted variables, including orthographic awareness, executive function, visual spatial perception, and character reading, and reading comprehension were shown in Table 2. Results showed that most measures in kindergarten were associated with Chinese character reading at grade 1, except for comprehension, inhibition and attention, Moreover, most measures in kindergarten were related to reading comprehension at grade 1, except for morphological awareness.

**Table 1:** Descriptive statistics and correlations among all variables.

	1	2	3	4	5	6	7	8	9	10	11	12	13	14	15	16	17
1.T1 Character reading	1																
2.T1 Listening comprehension	0.11	1															
3.Digit recall backward	0.24^*^	0.09	1														
4.Inhibition	0.20	0.13	0.15	1													
5.Sustained attention	0.22	0.19	0.20	0.42^***^	1												
6.Blocks	0.23^*^	0.22	0.25^*^	0.48^***^	0.34^**^	1											
7.Visuo-spatial sketchpad	0.11	0.27^*^	0.18	0.18	0.27^*^	0.36^**^	1										
8.Orientation	-0.10	-0.16	0.31^**^	0.25^*^	0.28^*^	0.32^**^	0.24^*^	1									
9.Digit recall forward	0.25^*^	0.02	0.33^**^	0.10	0.08	0.29^*^	0.19	0.13	1								
10.Syllable deletion	0.41^***^	0.10	0.35^**^	0.31^**^	0.29^*^	0.36^**^	0.24^*^	0.26^*^	0.31^**^	1							
11.Morphome construction	0.23^*^	0.16	0.15	0.15	0.06	0.46^***^	0.14	0.07	0.26^*^	0.30^**^	1						
12.Mirror characters	0.57^***^	0.12	0.26^*^	0.16	0.11	0.11	0.12	0.07	0.29^**^	0.34^**^	0.16	1					
13.Pseudo-characters	0.37^***^	0.14	0.12	0.22	0.27^*^	0.23^*^	0.15	0.10	0.07	0.21	0.06	0.43^***^	1				
14.Radicles	0.26^*^	0.03	0.14	0.23^*^	0.22	0.28^*^	0.25^*^	0.16	0.44^***^	0.27^*^	0.14	0.23^*^	0.24^*^	1			
15.Rapid automatized naming	-0.44^***^	-0.09	-0.37^***^	-0.11	-0.29^*^	-0.15	-0.14	-0.12	-0.10	-0.44^***^	-0.17	-0.33^**^	-0.16	0.05	1		
16.T2 Character reading	0.76^***^	0.13	0.27^*^	0.14	0.19	0.24^*^	0.18	0.25^*^	0.18	0.37^***^	0.33^**^	0.47^***^	0.22	0.23^*^	-0.47^***^	1	
17.T2 Reading comprehension	0.70^***^	0.24^*^	0.27^*^	0.29^*^	0.35^**^	0.24^*^	0.22	0.21	0.31^**^	0.43^***^	0.21	0.47^***^	0.25^*^	0.26^*^	-0.37^***^	0.70^***^	1
Mean	397.91	32.93	3.42	1291.41	640.76	27.72	4.12	8.21	7.75	14.52	26.37	48.94	36.04	9.65	31.42	740.33	29.11
*SD*	359.84	3.64	1.44	863.40	96.93	4.53	0.78	7.88	1.82	6.37	6.66	11.54	8.89	3.90	10.02	264.51	10.71
Skewness	0.56	-2.11	1.24	3.06	0.35	-2.00	-0.04	6.05	-0.43	-0.67	-1.34	-0.12	-0.84	-0.35	0.63	-0.85	0.26
Kurtosis	-1.32	8.89	3.14	10.95	-0.41	8.87	-0.98	44.03	2.35	-0.62	2.43	-0.28	2.20	-0.66	-0.21	-0.63	-0.13
Reliability	0.99	0.96	0.60	0.83	0.85	0.63	0.85	0.79	0.70	0.94	0.86	0.90	0.73	0.75	0.88	0.99	0.95


*^∗∗∗^*p* < 0.001, ^∗∗^*p* < 0.01, ^∗^*p* < 0.05. Variables 1–15 were measured at kindergarten. Variables 16–17 were measured at grade 1.*

**Table 2:** Correlations among word reading, reading comprehension, attention, morphological awareness, and phonological awareness and the compacted variables (executive function, visual spatial perception, orthographic awareness).

	1	2	3	4	5	6	7	8	9	10
1.T1 Character reading	1									
2.T1 Listening comprehension	0.14	1								
3.T1 Executive function	0.30^**^	0.17	1							
4.T1 Visual spatial perception	0.20	0.32^**^	0.46^***^	1						
5.T1 Attention	0.22	0.20	0.40^***^	0.41^***^	1					
6.T1 Morphological awareness	0.23^*^	0.21	0.21	0.31^**^	0.06	1				
7.T1 Phonological awareness	0.41^***^	0.12	0.43^***^	0.42^***^	0.22	0.34^**^	1			
8.T1 Orthographic awareness	0.56^***^	0.16	0.33^**^	0.30^**^	0.27^*^	0.16	0.44^***^	1		
9.T2 Character reading	0.76^***^	0.16	0.26^*^	0.30^**^	0.19	0.33^**^	0.34^**^	0.42^***^	1	
10.T2 Reading comprehension	0.70^***^	0.26^*^	0.36^***^	0.31^**^	0.35^**^	0.21	0.46^***^	0.45^***^	0.70^***^	1


*^∗∗∗^*p* < 0.001, ^∗∗^*p* < 0.01, ^∗^*p* < 0.05; T1: at kindergarten; T2: at grade 1.*

### Regression Analyses

The hierarchical linear regression with variables entered in *a priori* sequence was used to examine the unique cognitive predictors of individual differences when Chinese character reading and reading comprehension measures were used as the dependent variables, respectively.

Results showed that after controlling age, gender and character reading in kindergarten, rapid automatized naming and morphological awareness were uniquely associated with Chinese character reading (Table 3). As reading comprehension was not tested for kindergarteners considering difficulty level, character reading and listening comprehension were controlled as the substitute (Table 4). Results showed sustained attention was uniquely related to reading comprehension. Moreover, basic cognitive processing would not contribute to either character or reading comprehension when basic language processing was controlled.

**Table 3:** Autoregressive regression explaining Chinese character reading at Grade 1 with age, gender and character reading at kindergarten controlled.

		Chinese Character Reading at Grade 1			
	
Block and Variables		*β*	*T*	*R^2^*	*ΔR^2^*
Block 1	Age	-0.10	-1.24	0.01	0.01
	Gender	0.05	0.65	0.57	0.56^***^
Block 2	CR_K	0.67	6.60^***^		
Block 3	EF	-0.01	-0.07	0.59	0.02
	SA	-0.05	-0.58	0.64	0.05
	VSP	0.16	1.64		
Block 4	PA	-0.10	-1.05		
	MA	0.16	1.93^b^		
	OA	-0.06	-0.60		
	RAN	-0.20	-2.10^*^		
Block 3	PA	-0.10	-1.05	0.62	0.05
	MA	0.16	1.93^b^		
	OA	-0.06	-0.60		
	RAN	-0.20	-2.10^*^		
Block 4	EF	-0.01	-0.07	0.64	0.02
	SA	-0.05	-0.58		
	VSP	0.16	1.64		


*N = 82. EF, executive function; SA, sustained attention; VSP, visual spatial perception; PA, phonological awareness; MA, morphological awareness; OA, orthographical awareness; RAN, rapid automatized naming; CR_K, character reading at kindergarten. ^∗∗∗^*p* < 0.001, ^∗∗^*p* < 0.01, ^∗^*p* < 0.05, ^*b*^*p* = 0.055.*

**Table 4:** Autoregressive regression explaining reading comprehension at Grade 1 with age, gender, character reading, and listening comprehension in kindergarten controlled.

		Reading comprehension at Grade 1			
	
Block and Variables		*β*	*t*	*R^2^*	*ΔR^2^*
Block 1	Age	-0.03	-0.30	0.01	0.01
	Gender	0.08	0.96		
Block 2	CR_K	0.64	5.99^***^	0.53	0.53^***^
	LC	0.17	1.88		
Block 3	EF	0.09	0.85	0.59	0.06^*^
	SA	0.21	2.24^*^	0.61	0.02
	VSP	-0.03	-0.25		
Block 4	PA	0.18	1.72		
	MA	-0.04	-0.48		
	OA	-0.10	-1.00		
	RAN	0.04	0.44		
Block 3	PA	0.18	1.72	0.56	0.03
	MA	-0.04	-0.48	0.61	0.05
	OA	-0.10	-1.00		
	RAN	0.04	0.44		
Block 4	EF	0.09	0.85		
	SA	0.21	2.24^*^		
	VSP	-0.03	-0.25		


*N = 82. LC, listening comprehension; EF, executive function; SA, sustained attention; VSP, visual spatial perception; PA, phonological awareness; MA, morphological awareness; OA, orthographical awareness; RAN, rapid automatized naming; CR_K, character reading at kindergarten. ^∗∗∗^*p* < 0.001, ^∗∗^*p* < 0.01, ^∗^*p* < 0.05.*

### Mediation Analyses

**FIGURE 3:**
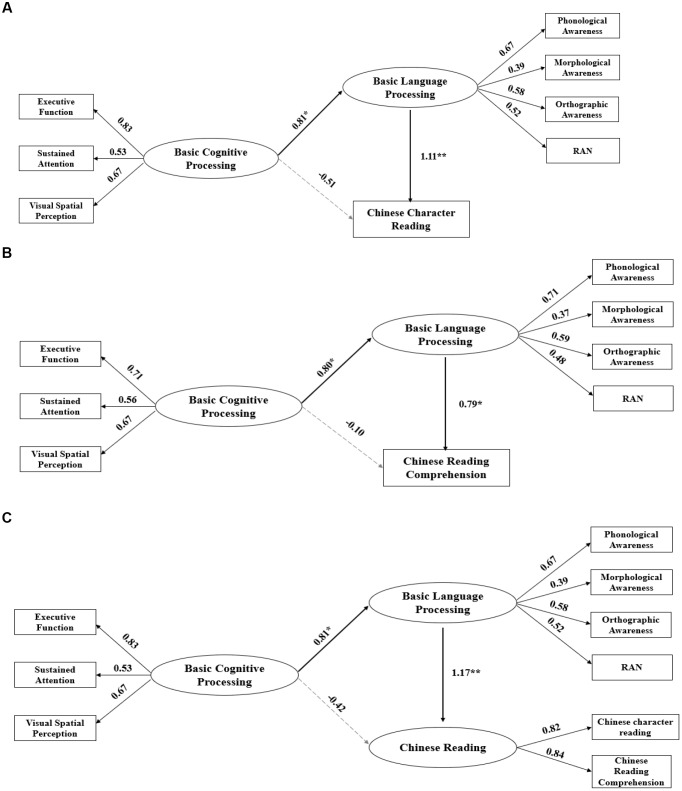
The mediation effect of basic language processing on the prediction of basic cognitive processing on Chinese character reading and reading comprehension. **(A)** Prediction of basic cognitive processing on Chinese character reading. Model fit: χ^2^ = 19.75, *df* = 18, RMSEA = 0.04, CFI = 0.99, NFI = 0.96, NNFI = 0.97, IFI = 1.00, SRMR = 0.03. **(B)** Prediction of basic cognitive processing on Chinese reading comprehension. Model fit: χ^2^ = 13.59, *df* = 18, RMSEA = 0.01, CFI = 1.00, NFI = 0.99, NNFI = 1.00, IFI = 0.99, SRMR = 0.02. **(C)** Prediction of basic cognitive processing on Chinese reading. Model fit: χ^2^ = 29.88, *df* = 24, RMSEA = 0.06, CFI = 0.97, NFI = 0.92, NNFI = 0.96, IFI = 0.93, SRMR = 0.06.

Here we would test our hypothesis that there might be a total indirect effect of the association between basic cognitive processing and reading, with the mediation of basic language processing. To achieve the goal, we included Chinese character reading as the dependent variable in the first model, reading comprehension as the dependent variable in the second model, and as reading includes both word recognition and comprehension, we included them as observed variables for children’s reading ability in the third model (Figure 3). Age and gender were no included in the model because of two reasons. First, children were at the same grade and gender was balanced to rule out their effects. Indeed, regression model showed age and gender made almost no contribution to children’s character reading and reading comprehension. Second, we only have a limited sample size to do the mediation model. Therefore, the two variables were not included. To explore the goodness of fit, a structural equation model (SEM) was specified using Lisrel 9.2 (Jöreskog and Sörbom, 2015). Missing data were dealt with using maximum likelihood estimation and all measure scores were standardized. The path coefficients for the mediation models are shown in Figure 1, where the significant paths are drawn with solid lines while the non-significant paths are drawn with dashed lines. Coefficients in the completely standardized solution might be larger than one (Jöreskog, 1999). The goodness of fit indexes including the ratio of the chi-square (χ^2^) to the degrees of freedom (*df*), root-mean-square error of approximation (RMSEA), comparative fit index (CFI), normed fit index (NFI), non-normed fit index (NNFI), incremental fit index (IFI), and standardized root-mean-square residual (SRMR) were shown in the figures. Results showed the mediation models fitted the data well and the mediation effects were satisfactory. It indicates that the predictions of basic cognitive abilities, no matter on future Chinese character reading, reading comprehension or compacted reading, were all mediated by basic language processing.

## Discussion

The present study extends earlier studies by highlighting how basic language processing and basic cognitive processing of kindergarten children contribute to Chinese character reading and reading comprehension at grade one. Results revealed that morphological awareness and RAN showed significant predictive effects on later Chinese character reading, whereas sustained attention and character reading in kindergarten were significantly associated with later reading comprehension. As expectedly, we also found the mediation effect of basic language processing between basic cognitive processing and Chinese reading. Below, we discuss these findings in detail.

Controlling the effects of basic cognitive processing and character reading performance at kindergarten, our study still found morphological awareness and RAN accounted for significant variance in later Chinese character reading. Beginning learners usually learn single-component characters, in which the semantic element facilitates children to understand the meaning of the character. When they learn compound characters, which are mostly comprised of two components, including a phonetic radical and a semantic radical. Young children will employ familiar semantic radicles of unfamiliar characters to distinguish analogical characters (Shu et al., 2006). McBride-Chang et al. (2003) also demonstrated morphological awareness predicted unique variance in Chinese character recognition abilities of beginning learners, after controlling for confounding factors. In addition, RAN has additionally been proven an important predictor of reading, which is consistent across English and Chinese (Compton, 2003; Pan et al., 2011). RAN reflects the rate of access to phonological information stored in long-term memory (Wagner and Torgesen, 1987). Similar to the process of Chinese single character recognition, RAN requires children to recognize and name a list of stimuli. The common underlying processes between RAN and Character reading seem to make RAN a promising predictor of early reading (Pan et al., 2011). It has been showed that the deficit in RAN limited children’s Chinese reading acquisitions (Ho et al., 2004; Liao et al., 2015; Peng et al., 2016).

Evidence showed that phonological awareness was consistently related to alphabetic language reading, while mixed relations were found in non-alphabetic language. Chinese differs from English and other alphabetic orthographies in that each Chinese character represents both a syllable and a morpheme. Children often acquire Chinese character reading by learning the semantic radical embedded within the characters, and less so via phonological processing (Shu and Anderson, 1997). Different from the alphabetic language system, the current study showed that phonological awareness was not uniquely associated with Chinese word learning. Shu et al. (2006) noticed that Chinese was relatively phonologically unpredictable compared to alphabetic languages. Among poor readers of Chinese, phonological awareness difficulties are sometimes a relatively minimal problem (Ho et al., 2004).

Reading comprehension is a complex process that involves both bottom-up character reading processes and top-down comprehension processes (e.g., Perfetti, 1999). Lam and Beale (1991) examined the relation between sustained attention and reading comprehension and found that sustained attention was significantly correlated with the reading comprehension scores. Children who were rated as suffering from high levels of inattention performed worse in the reading tests. This result was likely attributable to the fact that children had to identify every character and to connect the words together to comprehend. To finish the reading comprehension task, they needed to comprehend the meaning of the sentence and match it with several pictures and find the correct one. With these procedures in mind, sustained attention is undoubtedly essential. The present results were in line with the finding that reading comprehension problems are common in children and adolescents with Attention Deficit/Hyperactivity Disorder (ADHD; Stern and Shalev, 2013).

We found that character reading, rather than listening comprehension, explained significant variance in reading comprehension, indicating character reading/decoding had much overlap with early sentence reading comprehension (Rasinski, 2003). However, when controlling for gender and age, we did not find the effects of metalinguistic awareness and RAN, respectively, on reading comprehension. Perhaps these metalinguistic skills exercised most of their effects on character reading rather than on comprehension *per se*. Our results showed that for character reading, basic language processing explained more variance than basic cognitive processing while for reading comprehension, basic cognitive processing explained more variance than basic language processing. It may be that other specific skills that were not measured in the present study are more important for reading comprehension. For example, inference and grammatical skills are likely particularly useful for reading comprehension (e.g., Kendeou et al., 2014; Kim, 2017).

The mediation results were consistent with previous studies. For example, Facoetti et al. (2003) showed that attention might take effects on the development of phonological and orthographic representations that are essential for learning to read. In the mediation models, we did not include any reading performance in kindergarten because they covered the effects of basic cognitive and specific processing on later reading and explained most variance in later reading (57% in character reading and 53% in reading comprehension). In consistent with our hypothesis, the effect of basic cognitive processing on later reading was mediated by basic language processing. Results suggest that basic cognitive processing might advance children quickly and accurately acquire basic language abilities, such as RAN and word meaning, which in turn, would help children learn more advanced reading abilities (e.g., reading comprehension).

### Limitations

A couple of limitations of the present study should be mentioned when interpreting our findings. First, some advanced language factors were not included in our analysis that might be particularly important for reading comprehension, such as vocabulary and inference making as suggested in previous studies among English-speaking children (Kim, 2017). We indeed assessed vocabulary in our study, but the reliability of this measure based on the current sample is rather low (alpha < 0.50) and thus was excluded from the analysis. Due to the nature of reading comprehension measure and our sample characteristics (very young children), advanced cognitive skills such as inference making are not appropriate in this study. Nevertheless, our study included several key variables that are critical to character reading in Chinese. Future research should focus more on advanced language variables and more complicated reading comprehension measures among more capable readers. Second, we did not include intelligence in our present study, because intelligence is not often considered as an important predictor of early reading development (word level reading in particular) (Fletcher et al., 1994). Nonetheless, since intelligence often overlap with other cognitive skills, future studies may be needed to control for intelligence to further examine the unique contribution of different cognitive skills to early reading. Third, little is understood about how the two aspects of reading in Chinese (i.e., character reading and reading comprehension) promote one another. Given children in our sample are around 6 to 7 years old, their reading comprehension has much overlap with character recognition. A longer longitudinal study that spans from Chinese children’s learning to read (character reading) to reading to learn (reading comprehension) is needed in future work to expand understanding of specific aspects reading. Finally, we noticed that the mediation model for Chinese reading had a standardized coefficient larger than one, which may indicate multicollinearity among variables in the model (Deegan, 1978; Jöreskog, 1999). We took out RAN from the model and compared the results with the original models. The results showed that the mediation effect of the basic language processing on the relations of basic cognitive processing with Chinese reading remained, and the multicollinearity disappeared (Supplementary Figure S1). This is consistent with the debate about the nature of RAN, as RAN arguably taps both general cognitive and language abilities (Georgiou et al., 2013). For example, Ziegler et al. (2010) demonstrated that RAN is one component of phonological processing and it shared common variance with phonological awareness in their relations to word reading accuracy. In contrast, Geary (2011) considered RAN as an index of fundamental speed of encoding and information processing. Our models provided evidence that RAN may tap multiple linguistic and cognitive processes.

### Contributions

Despite these limitations, this study brings the converging knowledge of cognitive processes that contribute to Chinese beginning readers’ reading. Theoretically, our findings in early Chinese reading suggest the integration model of reading is language specific, in which different basic cognitive and basic language skills may be involved depending on different languages. For example, the non-significant effect of phonological awareness on Chinese reading demonstrated the different role of phonological awareness in varied languages. Also, our findings suggest that the future studies should further test the integration model by including more basic and advanced cognitive and language skills among more capable readers. Practically speaking, our findings suggest some basic cognitive (attention) and basic language skills (morphological awareness) as possible early candidates for identifying young children at risk for character reading and reading comprehension problems and for instruction/intervention on character reading and reading comprehension. That said, our findings are correlational in nature. Future intervention studies should further confirm our findings to provide more robust suggestion for early intervention and screening procedures that consider basic cognitive and language skills.

## Author Contributions

XY designed the experiments, collected, and analyzed the data, and wrote the manuscript. XM designed the experiments interface, discussed the data analyses, and commented on the manuscript. PP discussed the data analyses and commented on the manuscript. All authors approved the final manuscript for publication.

## Conflict of Interest Statement

The authors declare that the research was conducted in the absence of any commercial or financial relationships that could be construed as a potential conflict of interest.
