# Supplementary figures and images for: Contributions of Basic Cognitive Processing to Chinese Reading: The Mediation Effect of Basic Language Processing
Source: Front Psychol. 2019 Jan 8;9:2670. doi: 10.3389/fpsyg.2018.02670 (PMC6331404; doi:10.3389/fpsyg.2018.02670)

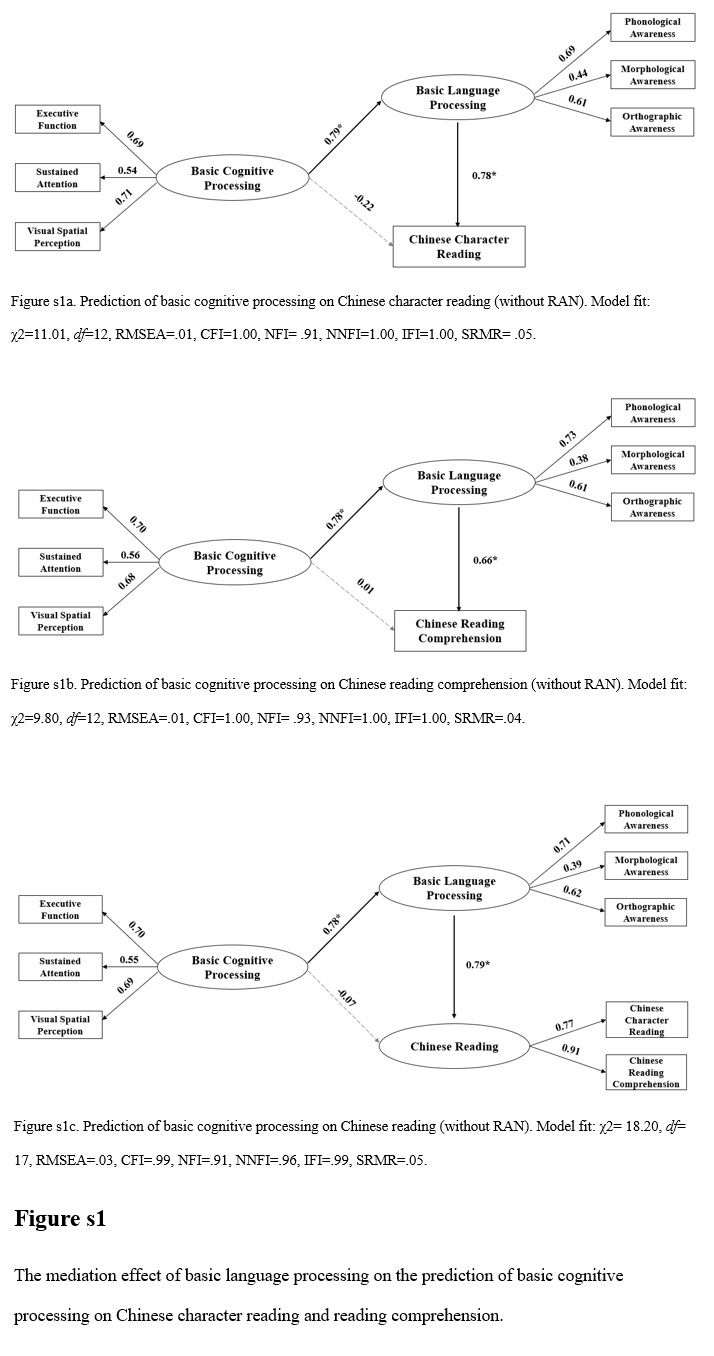

Supplement: Supplementary file 1 [file Image_1.jpg]
